# Hegar uterine dilator as an unusual cause of small bowel obstruction: a case report

**DOI:** 10.1186/1752-1947-4-208

**Published:** 2010-07-06

**Authors:** Chukwudi O Okorie

**Affiliations:** 1 Banso Baptist Hospital, Box 9, Kumbo, NWP, Cameroon

## Abstract

**Introduction:**

The unusual event of surgical instrument retention in the abdominal cavity usually occurs after laparotomy.

**Case presentation:**

A 45-year-old African woman from Cameroon with no previous abdominal surgery presented with a three-day history of colicky abdominal pain. Abdominal X-ray showed an opaque, linear object in the lower abdomen. Exploratory laparotomy revealed a Hegar uterine dilator that was lost during a dilatation and curettage performed seven years prior to the present admission.

**Conclusions:**

The trans-uterine route should be included as a rare and unusual source of surgical instruments retained in the abdomen.

## Introduction

Surgical instruments retained intra-abdominally usually occur after abdominal surgery and are straightforward to diagnose in conjunction with a history of previous laparotomy. Establishing the diagnosis pre-operatively of an abdominally retained surgical instrument in the absence of previous abdominal surgery will certainly be more challenging. Documenting and reporting all cases of retained surgical instruments not originating from previous abdominal exploration will help build a database and will educate physicians on how to avoid this dangerous medical error. From a literature search (Pubmed and Medline with no limitations until 2009) using search terms: abortion, Hegar uterine dilator, retained intra-abdominal surgical instruments, and bowel obstruction, no similar case reports were found. Hence, this case report documents the only known loss of a Hegar uterine dilator into the abdominal cavity during an abortion.

## Case presentation

**Figure 1:**
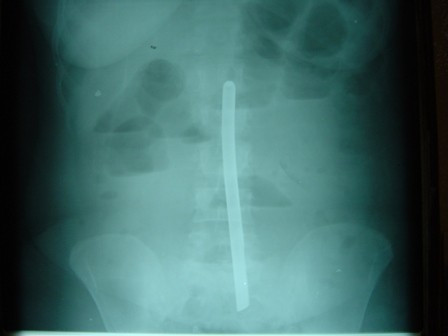
**Abdominal X-ray showing a linear, opaque object in the lower abdomen**.

A 45-year-old African woman from Cameroon presented with complaints of increasing generalized and colicky abdominal pain of three days duration. At the time of consultation, our patient reported that her last bowel movement was approximately 28 hours prior to presentation. Physical examination revealed a distended and tender abdomen with increased bowel sounds. There was no history of previous abdominal surgery, nor any visible abdominal wall scar. Her last menses was 12 days prior to presentation. Pelvic and rectal examinations revealed no abnormal findings. Her white blood count was 10,100/mm^3 ^with 73% neutrophils. Abdominal X-ray revealed a linear, opaque object in the lower abdomen, dilated small bowel loops, and air-fluid levels (Figure 1). Upon further questioning, there was no history of an anally inserted or swallowed object. The source of the opaque structure could not be determined by her history; therefore, with a diagnosis of partial bowel obstruction secondary to an intra-abdominal foreign body of unknown origin, our patient was prepared and taken to the operating room for an exploratory laparotomy.

**Figure 2:**
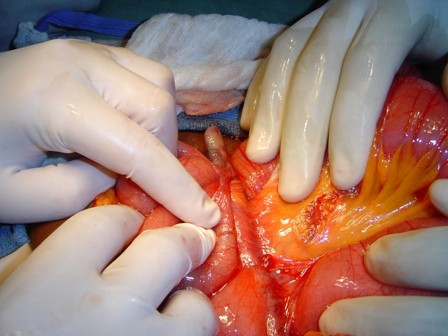
**Hegar uterine dilator encased in adhesions**.

**Figure 3:**
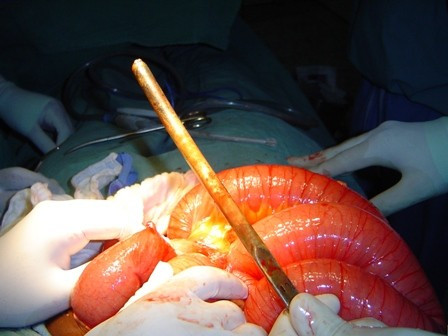
**Hegar dilator dissected out of adhesions**.

**Figure 4:**
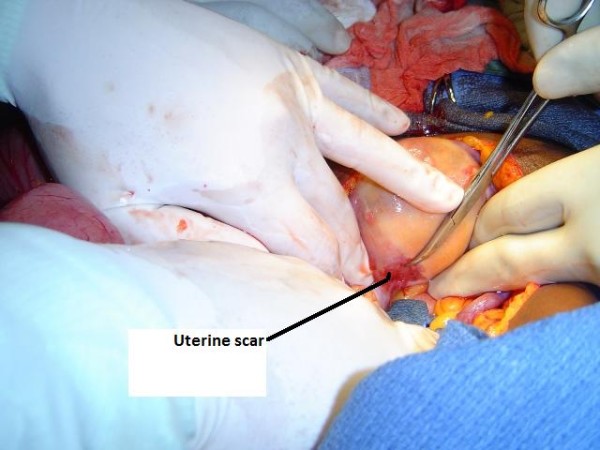
**Scarred area of old perforation at left lateral uterine wall**.

On abdominal exploration, a 10-gauge Hegar uterine dilator was dissected from multiple adhesions involving loops of small bowel (Figure 2 and 3). There was no bowel perforation. A scarred area was noted on the left posterior body of the uterus (Figure 4). The uterus was completely free of adhesions. On further questioning post-operatively, our patient revealed that she terminated a pregnancy seven years prior to presentation at an unspecified clinic.

## Discussion

Surgical instruments left in the abdominal cavity are an uncommon but dangerous surgical error [1] usually occurring during abdominal surgery. The presence of foreign bodies in the abdominal cavity can lead to the formation of adhesions [2], which are the most common cause of small bowel obstruction [3,4]. This case is unique in that the source of the intra-abdominal foreign body could not be determined or even imagined until surgery, and likewise our patient could not have related an abortion performed seven years prior to her present problem.

This case certainly calls for inclusion and early consideration of a trans-uterine source in the differential diagnosis of intra-abdominal foreign bodies including surgical instruments. As natural orifice trans-lumenal endoscopic surgery (NOTES) gains momentum, such cases may become more frequent.

## Conclusions

The trans-uterine route should be included as a rare and unusual source of surgical instruments retained in the abdomen. This case can assist in the future development of a classification system for various routes of abdominally retained surgical instruments, as this case demonstrates a source other than previous laparotomy.

## Consent

Written informed consent was obtained from the patient for publication of this case report and any accompanying images. A copy of the written consent is available for review by the Editor-in-Chief of this journal.

## Competing interests

The author declares that they have no competing interests.

## Authors' contributions

COO analyzed and interpreted the patient data, wrote and approved the manuscript.
